# SPOT-Contact-LM: improving single-sequence-based prediction of protein contact map using a transformer language model

**DOI:** 10.1093/bioinformatics/btac053

**Published:** 2022-02-01

**Authors:** Jaspreet Singh, Thomas Litfin, Jaswinder Singh, Kuldip Paliwal, Yaoqi Zhou

**Affiliations:** Signal Processing Laboratory, School of Engineering and Built Environment, Griffith University, Brisbane, QLD 4111, Australia; Signal Processing Laboratory, School of Engineering and Built Environment, Griffith University, Brisbane, QLD 4111, Australia; Signal Processing Laboratory, School of Engineering and Built Environment, Griffith University, Brisbane, QLD 4111, Australia; Signal Processing Laboratory, School of Engineering and Built Environment, Griffith University, Brisbane, QLD 4111, Australia; Institute for Glycomics, Griffith University, Southport, QLD 4222, Australia; Institute of Systems and Physical Biology, Shenzhen Bay Laboratory, Shenzhen 518055, China; Peking University Shenzhen Graduate School, Shenzhen 518055, China

## Abstract

**Motivation:**

Accurate prediction of protein contact-map is essential for accurate protein structure and function prediction. As a result, many methods have been developed for protein contact map prediction. However, most methods rely on protein-sequence-evolutionary information, which may not exist for many proteins due to lack of naturally occurring homologous sequences. Moreover, generating evolutionary profiles is computationally intensive. Here, we developed a contact-map predictor utilizing the output of a pre-trained language model ESM-1b as an input along with a large training set and an ensemble of residual neural networks.

**Results:**

We showed that the proposed method makes a significant improvement over a single-sequence-based predictor SSCpred with 15% improvement in the F1-score for the independent CASP14-FM test set. It also outperforms evolutionary-profile-based methods trRosetta and SPOT-Contact with 48.7% and 48.5% respective improvement in the F1-score on the proteins without homologs (Neff = 1) in the independent SPOT-2018 set. The new method provides a much faster and reasonably accurate alternative to evolution-based methods, useful for large-scale prediction.

**Availability and implementation:**

Stand-alone-version of SPOT-Contact-LM is available at https://github.com/jas-preet/SPOT-Contact-Single. Direct prediction can also be made at https://sparks-lab.org/server/spot-contact-single. The datasets used in this research can also be downloaded from the GitHub.

**Supplementary information:**

Supplementary data are available at *Bioinformatics* online.

## 1 Introduction

The past two decades have seen many developments in the field of protein structure prediction (Cheng *et al.*, 2019; Hanson *et al.*, 2020; Liu *et al.*, 2021). Significant headway has been observed specifically for protein secondary structure prediction and contact- and distance-map prediction (Fang *et al.*, 2018; Hanson *et al.*, 2019; Li *et al.*, 2019; Wang *et al.*, 2016; Wu *et al.*, 2020). These improvements have ultimately led to a considerable improvement in protein tertiary structure prediction, as observed in CASP13 (Cheng *et al.*, 2019).

Protein contact maps have been predicted by statistical inference based on Potts model and deep learning-based predictors. The predictors based on statistical inference are CCMpred (Seemayer *et al.*, 2014), Gremlin (Ovchinnikov *et al.*, 2014), EVFold (Sheridan *et al.*, 2015), plmDCA (Ekeberg *et al.*, 2014), FreeContact (Kaján *et al.*, 2014) and MetaPSICOV (Jones *et al.*, 2015). These methods were further improved by supervised deep learning-based methods such as RaptorX-Contact (Wang *et al.*, 2017), DeepCov (Jones and Kandathil, 2018), SPOT-Contact (Hanson *et al.*, 2018) and trRosetta (Wu *et al.*, 2020).

A common trait among these methods is the use of multiple sequence alignment (MSA) and other homology-based profile information. However, many proteins have very few or no homologs to generate MSA and homology profiles (Ovchinnikov *et al.*, 2017). In this case, their performance drops significantly (Chen *et al.*, 2020). Thus, it becomes essential to develop a method that predicts protein contact maps without using homologous information.

SSCpred (Chen *et al.*, 2020) is a recently published method that predicts contact maps using the one-hot encoding of the fasta sequence and the predicted one-dimensional structural properties of SPIDER3-Single (Heffernan *et al.*, 2018). The method uses a fully convolutional model with 30 ResNet blocks. The method performs adequately for proteins with few homologs but relatively poorer for those proteins with more effective homologs when compared to MSA-based techniques (Chen *et al.*, 2020). This limitation is expected as single-sequence-based method provides less information for the neural network to learn.

To improve the performance of single-sequence-based methods for the proteins with few homologs, there is a need for exploring other possible features beyond one-hot encoding. Recently, unsupervised deep learning methods were introduced to extract features inspired by Natural Language Processing’s language models (LM) (Elnaggar *et al.*, 2020; Heinzinger *et al.*, 2019; Rao *et al.*, 2019; 2020). These methods are trained on protein reference libraries such as UniRef (Suzek *et al.*, 2007), Uniclust (Mirdita *et al.*, 2017), Pfam (Bateman *et al.*, 2004) and BFD (Steinegger *et al.*, 2019b; Steinegger and Söding, 2018). Recently published protein LM ESM-1b trained on UniRef50 used a Transformer-34 model to generate unsupervised embedding and attention map (Rao *et al.*, 2020). ESM-1b’s embedding was further used to predict the secondary structure and its attention map to train a downstream contact map prediction. However, a single layer regression model may not fully utilize the capability of the LM. Using an attention map for contact map prediction is intuitive because of natural 2D mapping.

In this work, we examined the use of ESM-1b’s attention map as an input feature for our model to improve the contact-map prediction of our single-sequence-based method. We demonstrated that unsupervised learning features concatenated with one-hot encoding and SPOT-1D-Single’s outputs (Singh *et al.*, 2021b) outperform the single-sequence-based SSCpred and the MSA-based predictors for proteins with a low effective number of homologous proteins (Neff). We also showed that an ensemble of models trained through different training approaches and different feature combinations adds to this improvement.

## 2 Materials and methods

### 2.1 Datasets

The datasets obtained here are same as those used in SPOT-1D-Single (Singh *et al.*, 2021b). Briefly, to curate a dataset, we utilized the benchmark dataset prepared by ProteinNet (AlQuraishi, 2019). It consists of 50 914 proteins submitted to PDB before 2016 with high resolution (< 2.5 Å) crystal structures and clustered at sequence identity cut-off at 95% according to MMseqs2 tool (Steinegger and Söding, 2017). ProteinNet provides a number of datasets at different sequence identity cut-offs, but we chose the dataset with the sequence identity cut-off of 95% for training to obtain as much data as possible to harness the full capabilities of recent deep learning algorithms.

To efficiently validate models during training and minimize possible over-fitting, we separated 100 proteins from the ProteinNet set and compared their Hidden Markov Models generated by HHblits with the Hidden Markov Models of other proteins in the training dataset and validation set using HHblits. Any proteins, which had hits with these 100 validation proteins at an e-value cut-off of less than 0.1, were removed. This left us with the final 39 120 proteins for the training set. After removing any proteins with a length more than 500 from both the training and validation sets, the final training and validation sets have 34 691 and 88 proteins, respectively.

For independent testing and comparison, we downloaded all protein structures released between May 2018 and April 2020. As it can be insufficient to remove homologous sequences, we removed any potential homologs in the training set to the test data by comparing the Hidden Markov Models of all post-2018 proteins to the Hidden Markov Models of all pre-2018 proteins using the HHblits tool at an e-value cut-off of less than 0.1 (Steinegger *et al.*, 2019a). This led to 669 proteins as a stringent test set named SPOT-2018.

To test how predictors perform on de-novo proteins and proteins without homologs, we separated 46 proteins from SPOT-2018 which have Neff = 1 forming a test set called Neff1-2018. This provides a reliable, stringent and completely independent benchmark to compare the performance of different predictors on sequentially isolated proteins. Neff is calculated with respect to the reference Uniclust30 dataset (Published February 2020).

Apart from SPOT-2018 and Neff1-2018, we used an additional independent test set CASP14-FM. This test set includes 15 free modeling targets released at CASP14 (Liu *et al.*, 2021). Free modeling targets are those proteins without known structural templates in the protein databank at the time of release. Supplementary Table S1 provides a brief description of the test sets utilized in this study.

### 2.2 Input features

To train an ensemble of neural networks proposed in this method, we used multiple combinations of several features, including one-hot encoding of amino acids, the output of SPOT-1D-Single (Singh *et al.*, 2021b) and attention maps from ESM-1b (Rao *et al.*, 2020). One-dimensional features of one-hot encoding and the output of SPOT-1D-Single were converted into two-dimensional features using an outer concatenation. From SPOT-1D-Single, we obtained the probabilities of three-state-secondary-structure (SS3) and eight-state-secondary structure (SS8), Solvent Accessible Surface Area (ASA), Half-Sphere-Exposure (HSE) and protein backbone torsion angles *ψ*, *ϕ*, *θ* and *τ* (Singh *et al.*, 2021b). Please note that the training, validation and test sets used here were originated from the same sets in SPOT-1D-Single except for those proteins with their sequence lengths of more than 500 amino acid residues were removed in this work. Thus, the possibility of overtraining is avoided. Attention maps from ESM-1b were gathered by using all twenty attention heads from the last layer of the transformer as well as twenty attention heads from every layer of the ESM-1b model. For both cases, we symmetrized and applied average product corrections to the extracted attention maps as done by (Rao *et al.*, 2020).

### 2.3 Performance evaluation

The aim of this research is to predict which amino acid pairs in a protein are in contact. Following the standard CASP definition (Ezkurdia *et al.*, 2009), protein residues are considered to be in contact when there is an inter-residue distance of ≤8.0 Å between two *C_β_* atoms. A contact between two residues is classified into three types: long (at least 24 residues apart), medium (between 12 and 23 residues apart) and short (between 7 and 11 residues apart) ranges. For these three types of contacts, we calculated top L/10, L/5, L/2 and L/1 highest-ranked predictions in terms of precision. For further assessment in this work, we also calculated the overall F1-score, Matthews Correlation Coefficient (MCC) (Chicco and Jurman, 2020), Sensitivity, Area Under Curve of Precision–Recall Curve (AUC) and Area Under Curve of Receiver Operating Characteristic (ROC) of short-, medium- and long-range contacts, together. We also obtained the F1-score, MCC, sensitivity for our model and all other predictors at the maximum F1-score cut-off for the dataset.

### 2.4 Neural networks

Our deep neural network architecture was inspired by the success of the ResNet architecture in protein contact-map and RNA secondary structure prediction (Singh *et al.*, 2021a; Wang *et al.*, 2017). In this article, we use a 12 block ResNet, which is the maximum depth that we could train on the available GPU. Instead of using vanilla ResNet models, we used a recently published version of ResNet (Duta *et al.*, 2020). This improved version of ResNet was shown to perform better than vanilla and pre-act ResNet for both image and video-based tasks. Here, we applied this architecture for the inter-residue contact prediction problem.

**Fig. 1.**
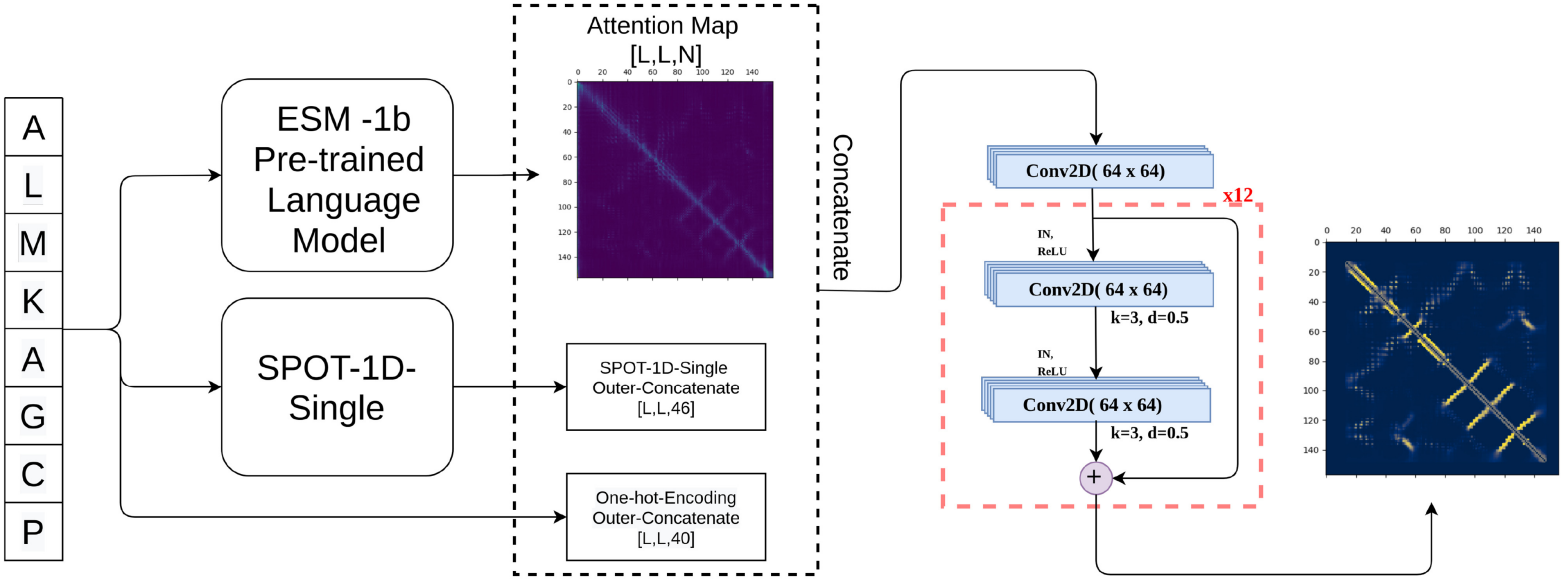
Overview of the model pipeline

As shown in Figure 1, we used convolutional layers with a channel size of 64 and kernel size of 3. We trained six models with the same architectural specifications but different input feature combinations as described in Table 1. The first three models in Table 1 were trained to predict the inter-residue contacts as a binary classification, while for the last three models, we predicted inter-residue distances as distance bins, and then we added the probabilities within the bins for the distances between 0 and 8 Å. 

**Table 1.** A description of feature combinations for the ensemble of trained models

Models	Features	Training strategy
Model1	Attention map (last layer)	Direct inter-residue contact prediction
Model2	Attention map (all layers)	Direct inter-residue contact prediction
Model3	Attention map (all layers) + one-hot encoding + SPOT-1D-Single	Direct inter-residue contact prediction
Model4	Attention map (last layer)	Inter-residue distance bin prediction
Model5	Attention map (all layers)	Inter-residue distance bin prediction
Model6	Attention map (all layers) + one-hot encoding + SPOT-1D-Single	Inter-residue distance bin prediction

The direct contact-map prediction models were trained using Binary Cross-Entropy loss, while the distogram-based prediction models were trained using Cross-Entropy loss. Apart from this major difference, other model hyperparameters and specifications are the same. This includes using the Adam optimizer with a learning rate of 0.001 and a batch size of 1. To avoid overfitting, all models were trained with early stopping of 3.

### 2.5 Method comparison

We compared SPOT-Contact-LM with LM’s supervised regression contact-map predictor ESM-1b, single-sequence-based SSCpred, profile-based-predictors trRosetta and SPOT-Contact. The above-stated methods trRosetta, SPOT-Contact and ESM-1b have stand-alone programs available online from https://github.com/gjoni/trRosetta, https://sparks-lab.org/server/spot-contact/ and www.github.com/facebookresearch/esm, respectively. Input to all profile-based methods including trRosetta was obtained from SPOT-Contact MSA generation pipeline for benchmarking purposes. For SSCpred, we utilized the web-server available online from http://csbio.njust.edu.cn/bioinf/sscpred/ due to lack of its stand-alone version.

## 3 Results

### 3.1 Feature importance

To understand the effect of different features, we trained a ResNet12 architecture on different input features and compared their performance on the test (SPOT-2018) set. For example, Table 2 shows that the model trained on the one-hot encoding of the fasta sequence only predicts the contact-map with top L/5 precision of 17% and 6% on medium- and long-range contacts. Adding the output of SPOT-1D-Single (a single-sequence-based predictor) to one-hot encoding improved the L/5 long range precision by 32% but only 3% for medium range contacts. By comparison, using the attention map output from the unsupervised learning method, ESM-1b significantly boosted the performance. The attention maps extracted from the last layer of the ESM-1b lead the L/5 precision to 31.8 and 30.3 on the medium and long-range contacts, respectively. These two results are 177% and 373% improvement over the model trained on SPOT-1D-Single + one-hot encoding. Using the attention maps extracted from all layers of ESM-1b further improves over using last layer only. Similar trends in terms of F1, MCC, precision and AUC of ROC are observed for using all ESM-1b attention maps as shown in Supplementary Table S2. As expected, concatenating all features together [one-hot encoding + SPOT-1D-Single + ESM-1B attention maps (all layers)] further showed a noticeable increase of 7.4% for L/5 precision of long-range contact over using ESM-1b attention maps only. Similar trends were observed for precision at other length cut-offs. Thus, a combination of one-hot encoding, SPOT-1D-Single and ESM-1b attention maps (all layers) was used for this work.

**Table 2.** Comparison of model precision by using ResNet12 trained on different feature combinations for long-range contacts on the SPOT-2018 test set

	Model	Medium range contacts				Long range contacts			
		L/10	L/5	L/2	L/1	L/10	L/5	L/2	L/1
1	One-hot encoding	20.79	17.39	13.04	9.98	7.02	6.15	5.04	4.40
2	One-hot encoding + SPOT-1D-Single	21.40	18.00	13.90	10.20	10.00	8.12	7.06	5.40
3	ESM-1b attention map (last layer only)	39.17	31.84	21.75	14.84	35.14	30.34	22.75	17.02
4	ESM-1b attention map (all layers)	40.03	32.83	22.49	15.26	36.03	30.92	23.75	18.13
5	All features	42.03	34.38	23.32	15.65	38.75	33.23	25.22	18.94

### 3.2 Direct versus distance contact-map prediction

To predict protein contact maps, we examined two different training strategies: direct contact-map prediction and distogram-based contact-map prediction by training a ResNet12 on one-hot encoding, SPOT-1D-Single’s output and ESM-1b’s attention maps concatenated together. Table 3 and Supplementary Table S3 shows that direct contact-map prediction performs slightly better, but the difference between the two training strategies is small. Thus, both strategies were used in different models for our final ensemble.

**Table 3.** Precision comparison of two training strategies: direct contact prediction, and distogram contact prediction for medium-, and long-range contacts on the SPOT-2018 set

Model	Medium range contacts				Long range contacts			
	L/10	L/5	L/2	L/1	L/10	L/5	L/2	L/1
Direct Contact Prediction	42.03	34.38	23.32	15.65	38.75	33.23	25.22	18.94
Distogram Contact Prediction	40.52	33.37	22.59	15.33	37.43	32.22	24.31	18.44

### 3.3 Ensemble learning performance

We further trained six different models with three best feature combinations using both distogram and direct contact prediction. We then ensembled the results of all six models to gain improvement over individual models by taking the mean of individual models. To understand the improvement gained, Table 4 presents the results of the selected six individual models and the ensemble of the six models on the validation set. The performance of the ensemble (SPOT-Contact-LM) is the highest among all individual models. For example, there is 2.1%, 3.3%, 2.8% and 3.5% improvement in precision over the best performing individual model for top L/10, L/5, L/2 and L long-range predictions, respectively. This performance gain is consistent across all other measures as shown in Supplementary Table S4.

**Table 4.** Comparison of individual model precision to the precision of the ensemble of models for long-range and medium-range contacts on the SPOT-2018 test set

Model	Medium range contacts				Long range contacts			
	L/10	L/5	L/2	L/1	L/10	L/5	L/2	L/1
Model1	39.17	31.84	21.75	14.84	35.14	30.34	22.75	17.02
Model2	40.03	32.83	22.49	15.26	36.03	30.92	23.75	18.13
Model3	42.03	34.38	23.32	15.65	38.75	33.23	25.22	18.94
Model4	38.52	31.53	21.86	14.85	35.32	30.19	22.80	17.20
Model5	40.16	32.85	22.13	14.93	37.34	31.82	23.87	17.77
Model6	40.52	33.37	22.59	15.33	37.43	32.22	24.31	18.44
Ensemble	42.43	34.41	23.63	15.88	39.60	34.35	25.94	19.62

### 3.4 Method comparison

**Fig. 2.**
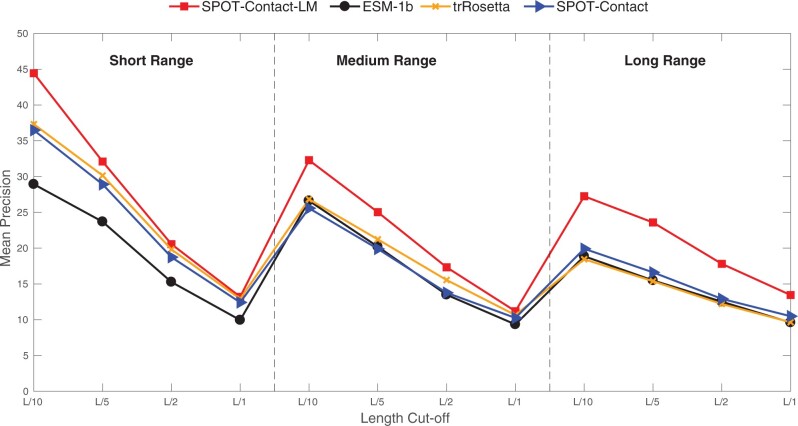
Precision-based comparison of SPOT-Contact-LM, SPOT-Contact, trRosetta and ESM-1b on Neff1-2018 for short-, medium- and long-range contacts

Because our method does not use MSA, it is of interest to compare all methods (MSA-based and single-sequence-based) on the proteins without homologous sequences (Neff = 1). Figure 2 compares SPOT-Contact-LM (this work) with ESM-1b (LM), SPOT-Contact (MSA-based) and trRosetta (MSA-based) for those proteins with Neff = 1 in the SPOT-2018 set (Neff1-2018). The evolution-based techniques (SPOT-Contact and trRosetta) achieve similar performance as ESM-1b with long-range L/5 precision around 16%. By comparison, the long-range L/5 given by SPOT-Contact-LM is 47% improvement with 24%. The improvement is consistently observed for other length cut-offs for medium- and long-range precision. Similar trends are also found across other performance measures, including F1-score, MCC, Sensitivity, AUC and ROC, as shown in Supplementary Table S5. To further confirm that the Neff1 proteins are not artifacts caused by a sequence library mismatch, we recalculated Neff for all protiens in Neff1-2018 using UniRef50 2018-03 release, the dataset that was used for training ESM-1b. Forty one proteins of the 46 proteins in the Neff1-2018 still have Neff = 1 and the trends are the same as shown in Supplementary Table S6.

The above comparison, however, is not made on the same network. To examine the impact of profile and LM-based features on the same network, we trained the single ResNet12’s model using the features used by SPOT-Contact and by PSSM and HMM alone. The models were trained following the same training strategy as SPOT-Contact-LM. The results of the models tested on Neff1-2018 is shown in Supplementary Table S7. Again, SPOT-Contact-LM significantly outperforms the models based on the features used by SPOT-Contact and by PSSM and HMM alone for the dataset of Neff1-2018.

**Fig. 3.**
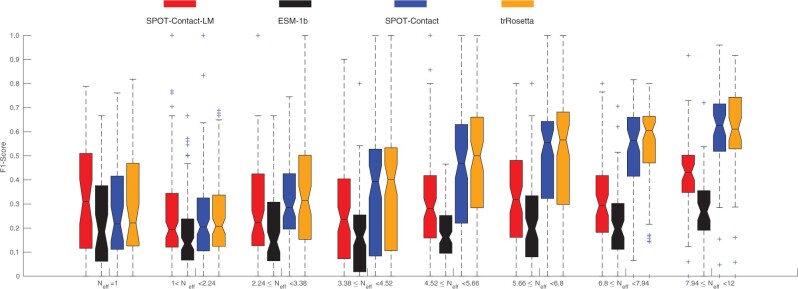
F1-score as a function of the number of effective homologous sequences (Neff) by SPOT-Contact-LM compared with other methods on SPOT-2018 for contact-map prediction

To illustrate the effect of homologous sequences, we plotted the F1-score of different predictors as a function of the Neff values in Figure 3. The performance of the profile-based predictors improves over SPOT-Contact-LM as Neff increases. In other words, SPOT-Contact-LM is not yet as competitive as evolution-based methods. This is because MSA of homologous sequence can provide co-mutation information more effectively than unsupervised learning.

**Fig. 4.**
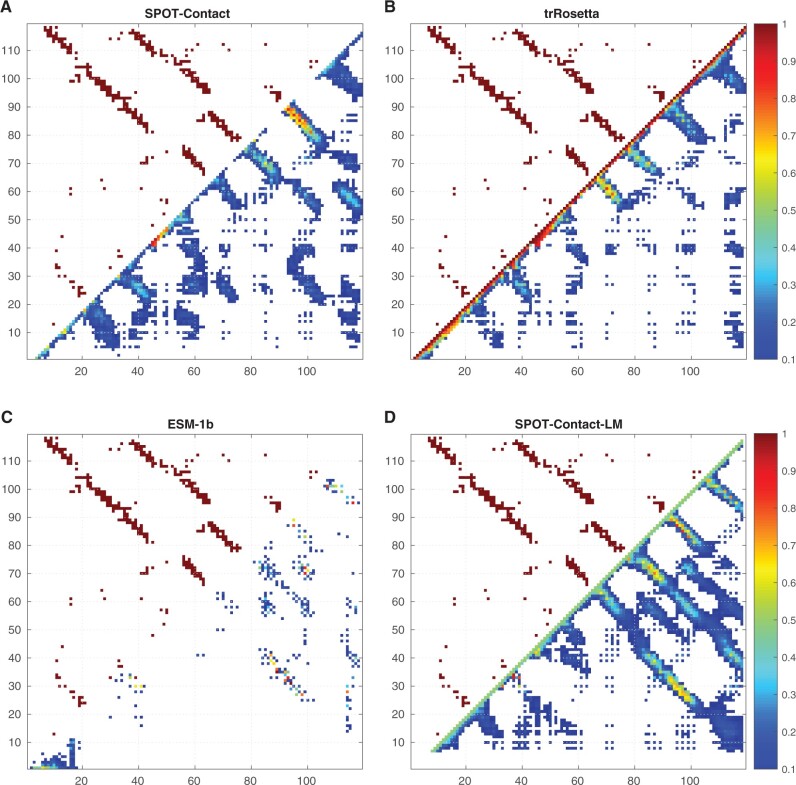
Comparison of the predictions for 5YKZ_A protein by four methods as labeled. The upper triangle and lower triangle represent the native and the predicted contact-map, respectively

The native and predicted contact-maps from SPOT-Contact-LM, SPOT-Contact, trRosetta and ESM-1b on an example protein (5YKZ_A) from Neff1-2018 are presented in Figure 4, which shows SPOT-Contact-LM provided a more accurate prediction of the contact-map for this low Neff protein, with the F1-scores of 0.215, 0.235, 0.252 and 0.388 for SPOT-Contact, trRosetta, ESM-1b and SPOT-Contact-LM, respectively.

### 3.5 Comparison with SSCpred

SSCpred is a single-sequence-based contact-map predictor that used the proteins released till 2019 April for training. To make a fair comparison, we compared SSCpred to other predictors on the CASP14-FM dataset. Table 5 shows that SPOT-Contact-LM performs much better than ESM-1b and SSCpred with long-range precision of 19%, 19%, 15% and 12% for length cut-offs of L/10, L/5, L/2 and L/1, respectively. These improvements are 50–110% better than SSCpred at different length cut-offs. The difference performance is smaller for medium-range. Supplementary Table S8 shows that the F1-score of SPOT-Contact-LM is higher than SSCpred.

**Table 5.** Precision-based comparison of SPOT-Contact-LM, SSCpred, ESM-1b and SPOT-Contact on the CASP14-FM set for medium and long range contacts

Model	Medium range contacts				Long range contacts			
	L/10	L/5	L/2	L/1	L/10	L/5	L/2	L/1
SPOT-Contact-LM	29.73	24.72	17.96	13.93	18.92	19.38	15.40	11.56
SSCpred	26.13	24.50	17.17	12.61	9.91	9.13	7.66	7.69
ESM-1b	22.97	19.82	15.58	10.85	17.12	12.47	9.42	7.38
SPOT-Contact (profile)	41.44	36.08	26.41	17.09	25.23	21.16	19.28	16.21

## 4 Discussion

In this article, we have developed a new protein contact-map predictor which uses the pretrained features from a transformer LM as input to predict contact maps without using homologous sequences. We used an ensemble of ResNet-based architectures trained on multiple combinations of several features and a large training set of almost 35 000 proteins with validation and test sets that are non-redundant to all training proteins according to HHsearch. The accuracy of SPOT-Contact-LM is higher than the evolutionary-profile-based SPOT-1D and trRosetta when the number of effective homologous sequence is low. This highlights that SPOT-Contact-LM can be used as a reasonably accurate screening tool for protein contact map prediction.

Using ESM-1b attention map in SPOT-Contact-LM makes it not possible to directly predict contact maps for proteins with more than 1024 amino acids. This should not prevent the use of SPOT-Contact-LM for large proteins because proteins are usually made of domains with less than 1000 residues.

A point of interest could be to profile our method (SPOT-Contact-LM) against a profile-based method (trRosetta) in terms of computational time. As shown in Supplementary Table S9, while running inference on CPU for CASP14-FM dataset of 15 proteins, SPOT-Contact-LM makes the prediction in 116 seconds which is 22 times faster than trRosetta. Also, on GPU, trRosetta took 1926 seconds which 42 times slower than SPOT-Contact-LM. As expected, the sequence profile generation takes significantly longer than the proposed method making the latter more suitable for genomic scale prediction.

Finally, SPOT-Contact-LM predicts the protein contact-map without using evolutionary features. The further improvement in protein contact-map prediction without evolutionary information may come from using more advanced architectural models such as Transformer (Vaswani *et al.*, 2017) or Performer (Choromanski *et al.*, 2020) for downstream supervised training.

## Supplementary Material

btac053_Supplementary_Material
