# Supplementary material for: SPOT-Contact-LM: improving single-sequence-based prediction of protein contact map using a transformer language model
Source: Bioinformatics. 2022 Feb 1;38(7):1888–94. doi: 10.1093/bioinformatics/btac053 (PMC9113311; doi:10.1093/bioinformatics/btac053)
Supplement: btac053_Supplementary_Material [file btac053_supplementary_material.pdf]

Supplementary Material: SPOT-Contact-LM: Improving Single-Sequence-Based Prediction of Protein Contact Map using a Transformer Language Model

Jaspreet Singh[1,*], Thomas Litfin[1], Jaswinder Singh[1], Kuldip Paliwal[1,*], and Yaoqi Zhou[2,3,4,*]

[1]Signal Processing Laboratory, School of Engineering and Built Environment, Griffith University, Brisbane, QLD 4111, Australia
[2]Institute for Glycomics, Griffith University, Parklands Dr. Southport, QLD 4222, Australia
[3]Institute for Systems and Physical Biology, Shenzhen Bay Laboratory, Shenzhen 518055, China
[4]Peking University Shenzhen Graduate School, Shenzhen 518055, P.R.China
[*]Correspondence to jaspreetsingh2@griffithuni.edu.au, K.paliwal@griffith.edu.au and zhouyq@szbl.ac.cn

Supplementary Table S1: A description of different test sets used in this research.

Dataset Name	Protein Count	Validation tool / Body	E-value Cutoff	Description	Average Neff
SPOT-2018	669	HHsearch	0.01	Post 2018 proteins independent to all pre-2018 proteins	4.47
Neff1-2018	46	HHsearch	0.01	SPOT-2018 subset of proteins with no homologs	1.0
CASP14-FM	15	CASP targets	-	Free modelling targets released during CASP-14	2.43

Supplementary Table S2: Comparison of ResNet12's model performance trained on different feature combinations on the SPOT-2018 set. We compared the F1-score, MCC, Sensitivity, Precision, AUC, and ROC for predicting all contacts (short-, medium-, and long-range).

	Feature	F1	MCC	Sensitivity	Precision	AUC	ROC
1	One-hot encoding	0.137	0.128	0.158	0.121	0.068	0.754
2	One-hot encoding + SPOT-1D-Single	0.169	0.148	0.168	0.14	0.1	0.768
3	ESM-1b attention map (last layer only)	0.282	0.278	0.249	0.324	0.219	0.842
4	ESM-1b attention map (all layers)	0.293	0.289	0.260	0.336	0.228	0.853
5	All features	0.301	0.296	0.276	0.331	0.245	0.868

Supplementary Table S3: Performance comparison of two training strategies: direct contact- and distogram contact-map prediction on the SPOT-2018 set.

Model	F1	MCC	Sensitivity	Precision	AUC	ROC
Direct Contact Prediction	0.301	0.296	0.276	0.331	0.245	0.868
Distogram Contact Prediction	0.299	0.294	0.268	0.337	0.234	0.861

Supplementary Table S4: Individual model performance as compared to the ensemble performance on the SPOT-2018 set for contact-map prediction.

Model	F1	MCC	Sensitivity	Precision	AUC	ROC
Model1	0.282	0.278	0.249	0.324	0.219	0.842
Model2	0.293	0.289	0.260	0.336	0.228	0.853
Model3	0.301	0.296	0.276	0.331	0.245	0.868
Model4	0.285	0.280	0.255	0.323	0.215	0.834
Model5	0.293	0.290	0.252	0.350	0.227	0.850
Model6	0.299	0.294	0.268	0.337	0.234	0.861
SPOT-Contact-LM	0.312	0.308	0.283	0.348	0.255	0.878

Supplementary Table S5: Comparison of SPOT-Contact-LM, SPOT-Contact, TrRosetta, and esm-1b on the Neff=1 set (Neff1-2018). To measure the performance of the predictors, we compared the F1-score, MCC, Precision, Sensitivity, AUC, and ROC of the overall prediction for all short-, medium-, and long-range predictions collectively, for the highest threshold of each predictor for this test set.

Model	F1	MCC	Sensitivity	Precision	AUC	ROC
SPOT-Contact-LM	0.3217	0.3156	0.2880	0.3643	0.2462	0.8431
ESM-1b	0.2156	0.2063	0.1994	0.2347	0.1367	0.7611
SPOT-Contact (profile)	0.2166	0.2101	0.1872	0.2569	0.1638	0.8221
TrRosetta (profile)	0.2163	0.2054	0.2155	0.2171	0.1059	0.7955

Supplementary Table S6: Comparison of SPOT-Contact-LM, SPOT-Contact, TrRosetta, and esm-1b on 41 proteins with Neff=1 from Neff1-2018 according to Uniref50 (2018-03 release). To measure the performance of the predictors, we compared the F1-score, MCC, Precision, Sensitivity, AUC, and ROC of the overall prediction for all short-, medium-, and long-range predictions collectively, for the highest threshold of each predictor for this test set.

Model	F1	MCC	Sensitivity	Precision	AUC	ROC
SPOT-Contact-LM	0.3019	0.2963	0.2667	0.3478	0.2244	0.8381
ESM-1b	0.2061	0.1979	0.1839	0.2344	0.1288	0.7548
SPOT-Contact (profile)	0.1952	0.1838	0.2026	0.1884	0.1419	0.8173
TrRosetta (profile)	0.1963	0.1853	0.2229	0.1755	0.0905	0.7878

Supplementary Table S7: Comparison of a single ResNet12's model performance when trained on evolutionary profiles (PSSM and HMM), SPOT-Contact features (PSSM, HMM, CCMPred, DCA, and SPIDER3) and ESM-1b output (SPOT-Contact-LM). To measure the performance of the predictors, we compared the F1-score, MCC, Precision, Sensitivity, AUC, and ROC of the overall prediction for all short-, medium-, and long-range predictions collectively, for the highest threshold of each predictor for the Neff1-2018 set.

Model	F1	MCC	Sensitivity	Precision	AUC	ROC
SPOT-Contact-LM (Single model)	0.283	0.277	0.252	0.324	0.213	0.793
PSSM and HMM	0.130	0.118	0.1839	0.129	0.131	0.683
SPOT-Contact Features	0.143	0.123	0.184	0.130	0.130	0.683

Supplementary Table S8: Comparison of SPOT-Contact-LM, SSCpred, ESM-1b, and SPOT-Contact on the CASP14-FM set. To measure the performance of the predictors, we compared the F1-score, MCC, Precision, Sensitivity, AUC, and ROC of the overall prediction for all short-, medium-, and long-range predictions collectively, for the highest threshold of each predictor for this test set.

Model	F1	MCC	Sensitivity	Precision	AUC	ROC
SPOT-Contact-LM	0.2061	0.1925	0.2069	0.2053	0.1322	0.7953
SSCpred	0.1797	0.1651	0.1943	0.1671	0.1088	0.7772
ESM-1b	0.1513	0.1372	0.1482	0.1546	0.0765	0.6776
SPOT-Contact (profile)	0.2813	0.2783	0.2316	0.3581	0.2384	0.8261

Supplementary Table S9: Inference time comparison of SPOT-Contact-Single and TrRosetta for prediction on 15 proteins of CASP14-FM.

Computational Specifications	TrRosetta	SPOT-Contact-Single
48 CPU threads on Intel(R) Xeon(R) CPU E5-2670 v3 @ 2.30GHz	2576 Seconds	131 Seconds
TITAN X (Pascal)	1926 Seconds	46 Seconds
